# Job‐related stress in psychiatric assistant nurses

**DOI:** 10.1002/nop2.103

**Published:** 2017-10-17

**Authors:** Hironori Yada, Hiroshi Abe, Hisamitsu Omori, Yasushi Ishida, Takahiko Katoh

**Affiliations:** ^1^ Department of Clinical Nursing Yamaguchi University Graduate School of Medicine Ube Yamaguchi Japan; ^2^ Faculty of Life Sciences Department of Public Health Kumamoto University Kumamoto Japan; ^3^ Department of Clinical Psychology Health Sciences University of Hokkaido Hokkaido Japan; ^4^ Faculty of Medicine Department of Psychiatry University of Miyazaki Miyazaki Japan

**Keywords:** assistant nurse, job stress, mental health, psychiatry

## Abstract

**Aim:**

We aimed to clarify how stress among psychiatric assistant nurses (PANs) differed from Registered Nurses (PRNs).

**Design:**

Cross‐sectional survey study was conducted with PRNs and PANs working in six psychiatric hospitals in Japan.

**Methods:**

The Psychiatric Nurse Job Stressor Scale (PNJSS) and the job stressor and stress reaction subscales of the Brief Job Stress Questionnaire measured stress in 68 PANs and 140 PRNs. The results were statistically analysed.

**Results:**

Psychiatric assistant nurses had significantly higher scores than PRNs on the job stressor subscales in psychiatric nursing ability, interpersonal relations and in the stress reaction subscales of irritability and somatic symptoms. “Psychiatric nursing ability,” “Communication” and “Use of techniques” were associated with almost all stress reactions in PANs than in PRNs.

## 1 INTRODUCTION

The nurse‐licensing scheme in Japan offers a Registered Nurse (RN) license and an assistant nurse license, which is similar to the scheme in other countries (Japanese Nursing Association 2014). There are different legal definitions of RN and assistant nurses in Japan. According to the Act on Public Health Nurses, Midwives and Nurses, “The term ‘Nurse’ as used in this Act means a person under licensure from the Minister of Health, Labour and Welfare to provide medical treatment or assist in medical care for injured and ill persons or puerperal women, as a profession.” In contrast, “The term ‘Assistant Nurse’ as used in this Act means a person under licensure from the prefectural governor to do as set forth in the preceding Articles under the direction of a physician, dentist, or Nurse, as a profession” (Japanese Law translation 2012, Ministry of Health, Labour and Welfare 2013). In other words, assistant nurses are specified as persons who provide nursing care under the supervision of not only physicians and dentists but also RNs. Assistant and RNs require different lengths of training, with a course term of 2 years for the former and 3 years for the latter, as a general rule (Ebihara, Takahashi, Ebihara, Satoh, & Sasaki,^2001^).

## 2 BACKGROUND

In Japan, there are 700,000 RNs and 150,000 assistant nurses employed as nursing staff (Ministry of Health, Labour and Welfare 2012a). However, this ratio is different in psychiatric wards where there are about 50,000 RNs and 33,000 assistant nurses (Ministry of Health, Labour and Welfare 2012a). The ratio of the assistant nurses to nursing staff in psychiatric wards is extremely high and a critical challenge is to examine the mental health care provided based on the characteristics of assistant nurses in addition to RNs in psychiatric wards.

As psychiatric treatment shifts from inpatient treatment to community care (Tachimori, Takeshima, Kono, Akazawa, & Zhao, 2015) and psychiatric nurses have an essential role in meeting the diverse mental health need (Pearson et al. 2015), team‐based care is better in improving patient satisfaction (Wen & Schulman, 2014). Assistant nurses are a member in team‐based care. In the absence of a RN, assistant nurses are sometimes required to act in their place (E‐Morris et al., 2010). They are required to carry out the same roles as nurses. However, assistant nurses earn roughly 20% less than the RNs in Japan (Ebihara et al., 2001). They may have difficulty achieving work satisfaction in the psychiatric care team.

In addition, RNs learn the nursing process of developing nursing care plans in Japan, while this process is not treated as compulsory education in the assistant nurse training. One study showed that the ability to develop nursing care plans differed significantly between assistant and RNs (Imai, 2001). The fact may increase difficulties in work. These conditions could result in job stress for assistant nurses.

Job‐related stress has a negative impact on patient care (Robinson, Clements, & Land, 2003) and may cause the assistant nurses to quit their jobs (Alexander, Lichtenstein, Oh, & Ullman, 1998); this, in turn, could decrease the quality of care. Reducing job‐related stress among assistant nurses is therefore an important occupational health task.

Although many studies have been conducted on stress among RNs and nursing students in general and psychiatric wards (Edwards & Burnard, 2003; McVicar, 2003; Pulido‐Martos, Augusto‐Landa, & Lopez‐Zafra, 2012), to our knowledge, no study has been conducted with a focus on job‐related stress in psychiatric assistant nurses (PAN). However, in a review of nursing staffs satisfactions, assistant nurses’ satisfactions with occupational status and autonomy as a professional was lower than in Registered Nurses (Nakagawa & Hayashi, 2004). Thus, this could result in stress among assistant nurses. Thus, PANs may have different unique job‐related stress with PRNs.

Identifying the factors that affect job‐related stress in assistant nurses could facilitate the provision of useful, informative, materials for self‐care and supervised care for assistant nurses in psychiatric wards.

One of the job‐related stress model is National Institute for Occupational Safety and Health (NIOSH) (Hurrell & McLaney, 1988). This model explained job‐related stress as stressors and stress reaction responses. Thus, in the analysing job‐related stress, we considered that it is necessary to evaluate from stressors and stress reactions. According to NIOSH, exposure to stressful working conditions (called job stressors) can have a direct influence on worker safety and health (NIOSH 1999). Individual and other situational factors can intervene to strengthen or weaken this influence (National Institute for Occupational Safety and Health, 1999). The aim of study was to clarify the stress as job‐related stressors and stress reactions of psychiatric assistant nurses (PANs) different from RNs (PRNs).

## 3 THE STUDY

### 3.1 Design

This cross‐sectional study included 385 PANs and PRNs from six psychiatric hospitals in Prefecture A in Japan; psychiatric nurses nursed patients with acute and chronic mental diseases, dementia, alcohol dependence, physical complications and adolescent mental diseases. In Japan, many psychiatric beds are in private hospitals and the ratio of nursing staff to patients was historically 5:1 (general hospital beds: 3:1) (Ministry of Health, Labour and Welfare 2012b), the number of beds per hospital ranged from roughly 40 to 300 in this study. Anonymous, self‐administered questionnaires were sent to participants via regular mail between 17 November and 21 December 2009. This paper reports a secondary analysis of data from 385 PANs and PRNs gathered in a previous study (Yada et al., 2011). However, the aims of the two studies were different. Among the 385 PANs and PRNs, 302 PANs and PRNs nurses provided written consent to participate in the study. Of these, data from 208 were included in the finally analysis; 58 incomplete questionnaires and 36 head and chief nurses were excluded (valid response rate: 54.0%).

### 3.2 Method

The survey recorded basic characteristics such as age, gender, experience of working in other departments and years of experience in psychiatry (basic attributes). Continuous variables were used to assess age and years of experience in psychiatry, while categorical variables were used to assess gender and experience of working in other departments. In job‐related stress model of the NIOSH, the concept of stress included stressors and stress reactions (Hurrell & McLaney, 1988). To assess job‐related stress in psychiatric nurses, we used the Psychiatric Nurse Job Stressor Scale (PNJSS) developed by Yada et al. (2011) and the Job Stressor (JSS) and Stress Reaction (SRS) scales from the Brief Job Stress Questionnaire (BJSQ) developed by Shimomitsu and Haratani (2000).

The PNJSS was developed to assess job‐related stressors to psychiatric nurses. The PNJSS comprised “psychiatric nursing ability” as knowledge and skills in psychiatric nursing, “attitude of patients” as degree of unpleasant patient attitude, “attitude towards nursing” as feeling in psychiatric nursing and “communication” as communication with patients and family of patients. Each item is ranked by the respondent along a 100‐mm visual analogue scale (VAS), with each 1 mm equal to 1 point. A higher score indicate a higher job stressor. As the reliability and validity of the PNJSS, The reliability was good [Cronbach’s α = 0.675–0.869, item‐scale correlation (*R*)= 0.265–0.570, *p *<* *.01 and test‐retest reliability (*R*)= 0.493–0.771, *p *<* *.01], the validity was also good [convergent validity (*R*)= 0.172–0.420, *p *<* *.01), predictive validity (*R*)= 0.201–0.453 and χ^*2*^/df ratio = 343.189/196, *p *<* *.01, goodness‐of‐fit index = 0.910, adjusted goodness‐of‐fit index = 0.883, comparative fit index = 0.924 and root mean square error of approximation = 0.050 as factorial validity] (Yada et al., 2011).

The BJSQ was developed by standardizing job‐related stress in multi‐industry. BJSS can measure stressors and stress responses in job‐related stress. The JSS measures job‐related stressors and assessed “quantitative overload,” “mental demand,” “physical workload,” “job control” as discretion in the workplace, “Use of techniques,” “interpersonal relations” as interpersonal relations among colleagues, “work environment” as physical environment in workplace, “fit to the job” and “reward of work.” The SRS measured psychological and physical stress reactions. The psychological stress reaction scale assessed lack of vigour, irritability, fatigue, anxiety and depressed mood, while physical stress reactions are assessed via a somatic symptoms subscale. Items are scored on a 4‐point scale. A higher score indicates a higher stress reaction. As the validity and the reliability of the BJSS, the factorial validity (the factor structure) of BJSS was confirmed by factor analysis. The reliability (Cronbach α) of the BJSQ was over 0.65 (Shimomitsu & Haratani, 2000).

### 3.3 Analysis

The mean, standard deviation (*SD*) and standard error (*SE*) were calculated for each continuous variable. Unpaired *t* tests and analysis of covariance (ANCOVA) were performed for inter‐group comparisons between continuous variables and chi‐squared tests were performed for inter‐group comparisons between categorical variables. The NIOSH job stress model proposed that stress reactions were affected by job stressors (Hurrell & McLaney, 1988). Pearson's correlation coefficients were calculated to reveal correlations between job‐related stressors and stress reactions both for PANs and PRNs. SPSS 21.0 for Windows (IBM, New York, USA) were used for statistical analyses. Statistical significance was set to 0.05.

### 3.4 Ethics

The researchers distributed questionnaire to the participants who were informed about the investigation's aim and their written consent was obtained. People were assured that their privacy would be protected, sealed completed questionnaire in an envelope provided in advance to ensure their anonymity. The Ethics Committee of the Kumamoto University Graduate School of Life Sciences approved this study protocol.

## 4 RESULTS

### 4.1 Characteristics of participants

Sixty‐eight participants were PANs and 140 were PRNs. When conducting the ANCOVA, we confirmed significant inter‐group distribution differences in basic attributes. To compare the basic attributes of PANs and PRNs, unpaired *t* tests were performed on age and years of experience in psychiatry, and chi‐squared tests were performed on gender and experience working in other departments. A significant difference in qualifications was seen in age and years of experience in psychiatry. The means of age and experience in psychiatry for PANs were significantly higher than those of PRNs, *p *<* *.01. The results are shown in Table 1.

**Table 1:** Demographic variable of participants

Variables	Assistant nurses (*N *=* *68)		Registered Nurses (*N *=* *140)		*t* or χ^*2*^ value	*p*‐value
	Mean or number	*SD* or %	Mean or number	*SD* or %		
Age	45.07	11.27	39.29	11.33	−3.457	.001
Gender						
Male (0)	19	27.9%	52	37.1%	1.724	.189
Female (1)	49	72.1%	88	62.9%		
Experience working in other department						
Yes (0)	50	73.5%	101	72.1%	0.044	.833
No (1)	18	26.5%	39	27.9%		
Years of experience in psychiatry department	15.22	11.47	10.12	8.89	−3.227	.002

Age and years of experience in psychiatric department were compared by *t* test. Gender and experience working in other departments were compared by χ^*2*^ value.

### 4.2 Qualification differences for stress subscale mean scores

We set age and years of experience in psychiatry (with a confirmed difference between PANs and PRNs) as covariates, calculated the mean and SE adjusted for PNJSS, JSS and SRS subscale scores and performed ANCOVAs. PANs had significantly higher scores than PRNs on the subscales for psychiatric nursing ability, interpersonal relations, irritability and somatic symptoms, *p *<* *.01. The results are shown in Table 2.

**Table 2:** Word differences for each stress subscale mean scores

Subscale	Assistant nurses (*N *=* *68)	Registered Nurses (*N *=* *140)	*F*‐value	*p*‐value
	Mean (*SE*)	Mean (*SE*)		
PNJSS				
Psychiatric nursing ability	58.27 (2.06)	51.09 (1.42)	7.989	.005
Attitude of patients	49.97 (2.38)	45.42 (1.64)	2.412	.122
Attitude towards nursing	71.58 (2.02)	69.01 (1.39)	1.059	.305
Communication	65.22 (3.36)	61.83 (2.32)	0.671	.413
JSS				
Quantitative overload	2.69 (0.08)	2.82 (0.05)	1.776	.184
Mental demand	2.96 (0.08)	2.97 (0.05)	0.015	.901
Physical workload	3.02 (0.12)	2.96 (0.08)	0.175	.676
Job control	2.64 (0.07)	2.53 (0.05)	1.576	.211
Use of techniques	2.39 (0.09)	2.30 (0.06)	0.627	.429
Interpersonal relations	2.38 (0.07)	2.21 (0.05)	4.011	.047
Work environment	2.68 (0.12)	2.53 (0.08)	1.033	.311
Fit to the job	2.43 (0.09)	2.24 (0.06)	2.893	.090
Reward of work	2.33 (0.10)	2.14 (0.07)	2.373	.125
SRS				
Lack of vigour	2.89 (0.08)	2.78 (0.06)	1.039	.309
Irritability	2.59 (0.10)	2.31 (0.07)	5.003	.026
Fatigue	2.65 (0.11)	2.50 (0.07)	1.286	.258
Anxiety	2.43 (0.09)	2.28 (0.06)	1.717	.192
Depressed mood	2.12 (0.09)	1.95 (0.06)	2.656	.105
Somatic symptoms	2.25 (0.08)	2.00 (0.05)	6.717	.010

Differences in mean scores (standard error) for each scale were analysed by analysis of covariance adjusted for age and years of experience in psychiatric department.

### 4.3 The correlations between job‐related stressors and stress reactions in PANs and PRNs

Significant positive correlations were seen with PANs and PRNs among almost all the subscales, *p *<* *.05. However, “Psychiatric nursing ability,” “Communication” and “Use of techniques” were associated with almost all stress reactions in PANs than in PRNs. The results are shown in Tables 3 and 4. Concretely, poorer psychiatric nursing abilities were associated with almost all of the stress reactions in PANs, while about PRNs, they were only associated with one reaction, lack of rigour. Communication difficulty were associated with almost all of the stress reactions in PANs, while about PRNs, they were only associated with two reaction, depressed mood and somatic symptoms. Use of techniques were associated with almost all of the stress reactions in PANs, while about PRNs, they were not associated with all stress reactions.

**Table 3:** Pearson's correlation coefficients between job‐related stressors and stress reactions of PANs

	Lack of vigour	Irritability	Fatigue	Anxiety	Depressed mood	Somatic symptoms
Psychiatric nursing ability	0.478**	0.357**	0.312**	0.241*	0.371**	0.120
Attitude of patients	0.330**	0.272*	0.516**	0.486**	0.505**	0.357**
Attitude towards	0.463**	0.535**	0.378**	0.271*	0.337**	0.117
Communication	0.306*	0.230	0.397**	0.208	0.257*	0.108
Quantitative overload	0.260*	0.344**	0.429**	0.473**	0.287*	0.192
Mental demand	0.083	0.266*	0.263*	0.445**	0.246*	0.230
Physical workload	−0.006	0.226	0.285*	0.359**	0.078	0.102
Job control	0.436**	0.357**	0.283*	0.216	0.280*	0.062
Use of techniques	0.473**	0.552**	0.469**	0.420**	0.430**	0.212
Interpersonal relations	0.379**	0.432**	0.106	0.099	0.235	0.241*
Work environment	0.366**	0.494**	0.416**	0.411**	0.431**	0.314**
Fit to the job	0.405**	0.233	0.242*	0.103	0.278*	0.053
Reward of work	0.462**	0.219	0.274*	0.095	0.342**	0.174

*p <* *.05, **p <* *.01.

**Table 4:** Pearson's correlation coefficients between job‐related stressors and stress reactions of PRNs

	Lack of vigour	Irritability	Fatigue	Anxiety	Depressed mood	Somatic symptoms
Psychiatric nursing ability	0.266**	0.155	0.129	0.064	0.145	0.004
Attitude of patients	0.157	0.378**	0.252**	0.282**	0.334**	0.349**
Attitude towards	0.426**	0.418**	0.385**	0.239**	0.298**	0.265**
Communication	0.126	0.151	0.134	0.118	0.173*	0.186*
Quantitative overload	0.082	0.273**	0.325**	0.401**	0.298**	0.298**
Mental demand	−0.010	0.072	0.197*	0.318**	0.245**	0.281**
Physical workload	0.039	0.215*	0.331**	0.343**	0.212*	0.224**
Job control	0.430**	0.175*	0.264**	0.227**	0.292**	0.238**
Use of techniques	0.154	0.024	0.153	0.126	0.163	0.111
Interpersonal relations	0.343**	0.428**	0.358**	0.391**	0.457**	0.412**
Work environment	0.260**	0.400**	0.236**	0.104	0.197*	0.289**
Fit to the job	0.467**	0.379**	0.384**	0.217**	0.366**	0.229**
Reward of work	0.465**	0.383**	0.360**	0.097	0.356**	0.257**

*p <* *.05, **p <* *.01.

## 5 DISCUSSION

In the present study on job‐related stress among PANs, we showed that job stress that is characteristic of PANs is related to psychiatric nursing ability and interpersonal relations and stress reactions are related to irritability and somatic symptoms. These findings are significantly higher in PANs than in PRNs. Moreover, “Psychiatric nursing ability,” “Communication” and “Use of techniques” were associated with almost all stress reactions in PANs than in PRNs. The following is a discussion of the results.

### 5.1 Examination of job‐related stress among PANs

Job stressors related to psychiatric nursing abilities were significantly higher in PANs than in PRNs and these could cause an increase in irritability. Individuals with schizophrenia or other psychiatric disorders often have psychiatric symptoms such as hallucinations and delusions (Gray & Roth, 2007) and nurses are at a high risk of violence from psychiatric patients (Zeng et al., 2013). Education for mental illness is required (Patrick & Amy, 2002). However, looking at nursing education, PANs have less course time than PRNs in the specialized subjects of psychiatric care and psychiatric nursing care practice (Japanese Nursing Association 2009). A deficient education prevents sufficient understanding of psychiatric patients and nursing practice among PANs, and it could increase “Psychiatric nursing abilities” and “irritability.”

As job‐related stressors related to nursing practice skills of PANs, we observed that “Psychiatric nursing ability,” “Communication” and “Use of techniques” were associated with almost all stress reactions in PANs than in PRNs. In recent years, the role and the service demanded of nurses have become diverse and complex with the advance in medical system. As mentioned above, PANs have less course time than PRNs. PANs cannot respond to social conditions. They particularly may have stress reactions on job‐related stressors with nursing practice skills as “Psychiatric nursing ability,” “Communication” and “Use of techniques.”

Meanwhile, PANs had significantly higher job stressors related to interpersonal relations than PRNs, which may heighten stress reactions such as irritability and somatic symptoms. In Japan, assistant nurses earn roughly 20% less than the RNs (Ebihara et al., 2001). However, they are required by hospitals to carry out the same roles as nursing staff (Ebihara et al., 2001). PANs may have conflicts to earn the low wages while being required for the same amount of work. This could increase their dissatisfaction with interpersonal relations at the workplace.

### 5.2 Future mental health care for PANs

The NIOSH job stress model shows that job‐related stress can be reduced by strengthening individual factors. Individual factors with skill and wages can be raised by educational involvement for PANs. As a measure to facilitate career advancement from assistant to RNs, in Japan, greater opportunities have been available since 2004 for assistant nurses to continue their studies in 2‐year correspondence courses while continuing to work (Yabuuchi, 2013). Career advancement by assistant nurses may not only raise nursing skills but also make for wage raise. Improvement on nursing skills will improve nursing abilities and wage raise will improve interpersonal relations. It may reduce many stress reactions for PANs. A support system needs to be developed for the workplace to facilitate career advancement of assistant nurses.

### 5.3 Limitations

A limitation of this study was its cross‐sectional design. Furthermore, having a sample of 208 psychiatric nurses (68 PANs and 140 PGNs) from Prefecture A and that too analysed 7 years ago could have introduced a bias. The questionnaire used in the present study did not include a scale related to job support and individual factors as characters and recognitions, as some factors may have a buffering effect on job stress. In future studies, the sample size should be increased by conducting a national investigation. Moreover, buffering effects should also be discussed in cross‐sectional designs.

## 6 CONCLUSION

We examined job‐related stress in PANs and found that PANs had significantly higher scores than RNs on the job‐related stressor subscales for psychiatric nursing ability and interpersonal relations, as well as higher scores on the stress reaction subscales on irritability and somatic symptoms. Further analysis suggested “Psychiatric nursing ability,” “Communication” and “Use of techniques” were associated with almost the stress reactions in PANs than in PRNs. Future challenges include the following creating support systems to facilitate career advancement of assistant nurses. Career advancement by assistant nurses may not only raise nursing skills but also make for wage raise. Improvement on nursing skills will improve nursing abilities and wage raise will improve interpersonal relations. It may reduce many stress reactions for PANs.

## CONFLICT OF INTEREST

No conflict of interest has been declared by the authors.
